# Iterative Immunostaining and NEDD Denoising for Improved Signal-To-Noise Ratio in ExM-LSCM

**DOI:** 10.21769/BioProtoc.5072

**Published:** 2024-09-20

**Authors:** Lucio Azzari, Minnamari Vippola, Soile Nymark, Teemu O. Ihalainen, Elina Mäntylä

**Affiliations:** 1 Tampere Microscopy Center (TMC), Tampere University, Tampere, Finland; 2 BioMediTech, Faculty of Medicine and Health Technology, Tampere University, Tampere, Finland; 3 Tampere Institute for Advanced Study, Tampere University, Tampere, Finland

**Keywords:** Iterative indirect immunofluorescence staining, Signal-to-background ratio, Expansion microscopy, Signal processing, Denoising

## Abstract

Expansion microscopy (ExM) has significantly reformed the field of super-resolution imaging, emerging as a powerful tool for visualizing complex cellular structures with nanoscale precision. Despite its capabilities, the epitope accessibility, labeling density, and precision of individual molecule detection pose challenges. We recently developed an iterative indirect immunofluorescence (IT-IF) method to improve the epitope labeling density, improving the signal and total intensity. In our protocol, we iteratively apply immunostaining steps before the expansion and exploit signal processing through noise estimation, denoising, and deblurring (NEDD) to aid in quantitative image analyses. Herein, we describe the steps of the iterative staining procedure and provide instructions on how to perform NEDD-based signal processing. Overall, IT-IF in ExM–laser scanning confocal microscopy (LSCM) represents a significant advancement in the field of cellular imaging, offering researchers a versatile tool for unraveling the structural complexity of biological systems at the molecular level with an increased signal-to-noise ratio and fluorescence intensity.

Key features

• Builds upon the method developed by Mäntylä et al. [1] and introduces the IT-IF method and signal-processing platform for several nanoscopy imaging applications.

• Retains signal-to-noise ratio and significantly enhances the fluorescence intensity of ExM-LSCM data.

• Automatic estimation of noise, signal reconstruction, denoising, and deblurring for increased reliability in image quantifications.

• Requires at least seven days to complete.

## Graphical overview


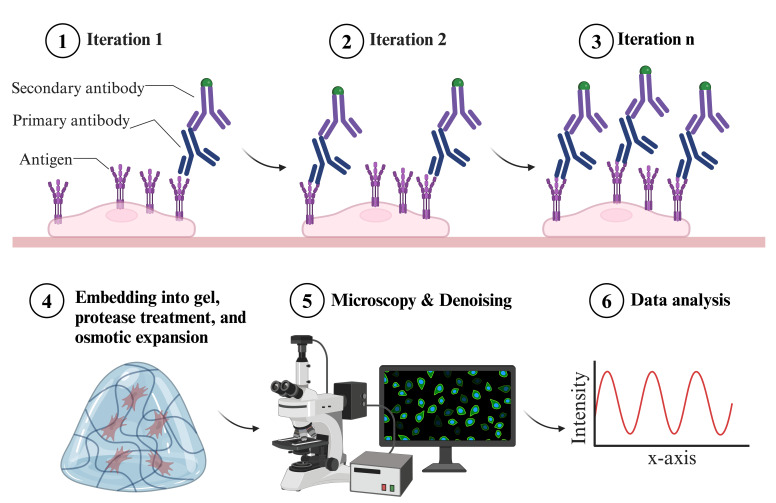


**Overview of indirect iterative immunofluorescence staining (IT-IF) procedure**

## Background

Expansion microscopy (ExM) has emerged as a tempting super-resolution microscopy technique for fluorescence imaging of cell and tissue organization. Along with other super-resolution modalities, ExM now extends optical imaging to the nanoscale, yielding unprecedented biological insights within molecularly crowded environments such as the nucleus. In ExM, fixed and gel-embedded samples are isotropically expanded in water by 4× to 10×, allowing imaging beyond the classical diffraction limit of light microscopy and resolving structures in near-molecular resolution. As a result, ExM allows a lateral resolution of ~70 nm with laser scanning confocal microscope (LSCM); the more recently developed iterative ExM including a second gel step allows 10× improvement, achieving a resolution of ~25 nm [2]. Recently, ExM has also been optimized for clinical specimens. The expansion pathology has been speculated to enable routine nanoscale imaging of pathological samples aiding clinical research [3].

However, ExM imaging of immunofluorescence-based samples has drawbacks related to technical difficulties in obtaining the optimal density of the fluorophores. ExM imaging of the samples results in a substantially weaker signal due to the dilution of the fluorophores caused by the sample expansion; the dilution scales to the third power of the (linear) expansion factor. Thus, expansion significantly reduces image brightness and creates noisy images with low signal-to-noise ratio (SNR). Conventional indirect immunostaining can be insufficient, leading to low-density labeling of the targets and, depending on the antibody type, often includes marked off-target staining of the background, also contributing to low image contrast and signal-to-background ratio (SBR) [4,5]. The accessibility of the target epitopes can be reduced, further lowering the image quality [6,7]. Also, super-resolution imaging methods pushing this barrier, such as structured illumination microscopy, pose challenges to the sample properties. When imaging biological samples in the sub-diffraction scale, low labeling density can lead to long acquisition times, higher excitation laser powers, and higher detector sensitivities, leading to increased noise and photobleaching.

We addressed these problems and developed an indirect iterative immunostaining (IT-IF) method for fluorescent labeling of ExM samples. The IT-IF counteracting the expansion-induced decrease in sample fluorescent intensity and signal quality significantly improves the sufficient staining of molecular structures aiding in structural analyses. We argue that the IT-IF approach facilitates nanoscopy, the detection and quantitative analysis of sub-resolution-sized molecular structures, especially within the nucleus. We show how post-image processing through noise estimation, denoising, and deblurring (NEDD) improves the low signal quality in ExM [8] even better than conventional deconvolution and provide step-by-step instructions on how to use the NEDD image processing tool to perform noise reduction on LSCM data. We prove that IT-IF leads to increased signal intensity without compromising the SBR, advancing super-resolution imaging of highly compact intranuclear structures. Finally, we exploit these methods to reveal nanoscopic structural details of nuclear lamina network organization.

## Materials and reagents

**Reagents**

1. Paraformaldehyde (PFA), 20% (Electron Microscopy Sciences, catalog number: 15713-S)
2. Primary antibody used in this study: Mouse anti-Lamin A/C (E-1) (Santa Cruz Biotechnology, catalog number: sc-376248)
3. Secondary antibody used in this study: Alexa^TM^-conjugated goat anti-mouse IgG (H+L) cross-adsorbed secondary antibody (Alexa Fluor^TM^ 488) (Thermo Fisher Scientific, catalog number: A-11008)
4. Triton^TM^ X-100 (Sigma-Aldrich, Merck, catalog number: X100-100ML)
5. Bovine serum albumin (BSA) (PAN Biotech, catalog number: P06-139210)
6. Sodium acrylate (Sigma-Aldrich, Merck, catalog number: 408220)
7. Acrylamide (0.40 g/mL, 40%) (Bio-Rad Laboratories, catalog number: 1610140)
8. N,N′-Methylenebisacrylamide (Sigma-Aldrich, Merck, catalog number: 1015460100)
9. Sodium chloride (Sigma-Aldrich, Merck, CAS #7647-14-5)
10. N,N,N′,N′-Tetramethylethylenediamine (TEMED, 100%) (Sigma-Aldrich, Merck, catalog number: 411019)
11. Acryloyl X – SE (10 mg/mL in dry DMSO) (Thermo Fisher Scientific, catalog number: A20770)
12. 4-Hydroxy-TEMPO (Sigma-Aldrich, Merck, catalog number: 176141)
13. Tris base (Sigma-Aldrich, Merck, catalog number: 10708976001)
14. Proteinase K (New England Biolabs, catalog number: P8107S)
15. Ammonium persulphate (APS, powder) (Sigma-Aldrich, Merck, catalog number: A3678)
16. High-melt agarose (1%, w/v) (Thermo Fisher Scientific, catalog number: J61123.22)
17. Phosphate buffered saline, with Ca^2+^/Mg^2+ ^(PBS, 10×) (Gibco, Thermo Fisher Scientific, catalog number: J62036.K2)
18. EDTA, 10 mM (Promega, catalog number: A2631)
19. Guanidine HCl (8 M) (Thermo Fisher Scientific, catalog number: 24115)
20. dH_2_O
21. Absolute EtOH

**Solutions**

1. 1× PBS (see Recipes)
2. Permeabilization solution A (see Recipes)
3. Permeabilization solution B (see Recipes)
4. Antibody diluent containing blocking agent (see Recipes)
5. Tris-HCl, 50 mM, pH 8 (see Recipes)
6. Sodium chloride, 5 M (see Recipes)
7. APS, 10% (w/v) (see Recipes)
8. Monomer solution (see Recipes)
9. Gelling solution (see Recipes)
10. Anchoring solution (see Recipes)
11. Digestion solution (see Recipes)

**Recipes**

1. **1× PBS**
  **Table:** 

Reagent	Final concentration	Quantity or Volume
10× PBS	1/10	10 mL
dH_2_O	n/a	90 mL
Total	n/a	100 mL
2. **Permeabilization buffer A**
  **Table:** 

Reagent	Final concentration	Quantity or Volume
1× PBS	n/a	50 mL
BSA* Triton X-100	0.5% (w/v) 0.5% (v/v)	0.25 g 250 µL
Total	n/a	50 mL
  *Prepare fresh. The prepared solution can be stored at 4 °C and used for two weeks.
3. Permeabilization buffer B*
  **Table:** 

Reagent	Final concentration	Quantity or Volume
1× PBS	n/a	50 mL
BSA**	0.5% (w/v)	0.25 g
Triton X-100	0.2% (v/v)	100 µL
Total	n/a	50 mL
  *Permeabilization buffer B contains a reduced concentration of Triton X-100 for a gentler permeabilization during iteration steps 2–4.
  **Prepare fresh. The prepared solution can be stored at 4 °C and used for two weeks.
4. **Antibody diluent containing blocking agent**
  **Table:** 

Reagent	Final concentration	Quantity or Volume
1× PBS	n/a	50 mL
BSA	3% (w/v)	1.5 g
Total	n/a	50 mL
5. **Tris-HCl, 50 mM, pH 8**
  **Table:** 

Reagent	Final concentration	Quantity or Volume
Tris base	50 mM	0.606 g
dH_2_O	n/a	60 mL
Adjust to pH 8.0 with HCl		
dH_2_O	to 100 mL	
Total	n/a	100 mL
6. **Sodium chloride, 5 M**
  **Table:** 

Reagent	Final concentration	Quantity or Volume
Sodium chloride	5 M (0.292 g/mL)	2.922 g
dH_2_O	to 10 mL	
Total	n/a	10 mL
7. **APS, 10% (w/v)**
  **Table:** 

Reagent	Final concentration	Quantity or Volume
APS powder*	10% (w/v)	1 g
dH_2_O	to 10 mL	
Total	n/a	10 mL
  *Store at room temperature (RT) in a desiccator.
8. Monomer solution*
  **Table:** 

Reagent (stock concentration)	Final concentration	Volume
Sodium acrylate (0.38 g/mL)**	0.086 g/mL	2.25 mL
Acrylamide (0.40 g/mL, 40%)	0.025 g/mL (2.5%)	0.58 mL
N,N′-Methylenebisacrylamide (0.02 g/mL, 2%)	0.0015 g/mL (0.15%)	0.75 mL
Sodium chloride (0.292 g/mL, Recipe 6)	0.117 g/mL	4 mL
10× PBS	1×	1 mL
dH_2_O	n/a	0.82 mL
Total	n/a	9.4 mL***
  *Can be stored at -20 °C for up to 2 months.
  **Sodium acrylate may have a variable purity affecting performance. Ensure it appears colorless under room light. If the reagent appears yellow, discard the stock without using it. Store at -20 °C in a desiccator.
  ***9.4/10 mL (1.06×), the remaining 6% of the volume will be brought up by the initiator, accelerator, and inhibitor as needed (see below).
9. Gelling solution*
  **Table:** 

Reagent	Volume
Monomer solution**	188 µL
dH_2_O (or inhibitor 4-Hydroxy-TEMPO)***	4 µL
TEMED****	4 µL
APS (10%, w/v) (Recipe 7)	4 µL
Total	200 µL
  *Prepare fresh and pipette reagents in the given order on ice.
  **Degas the monomer solution after preparation by using a degassing chamber to remove any air bubbles. Air will impair the homogenous polymerization of the gel.
  ***OPTIONAL replacement of water. 4-Hydroxy-TEMPO solution appears yellow. It slows down the gelling, giving a wider temporal working window.
  **** N,N,N′,N′-Tetramethylethylenediamine. Store at RT in a desiccator.
10. **Anchoring solution**
  **Table:** 

Reagent (stock concentration)	Volume
Acryloyl X – SE (10 mg/mL)	20 µL
PBS (1×)	1980 µL
Total	2,000 µL (2 mL)
  Prepare fresh.
11. **Digestion solution**
  **Table:** 

Reagent (stock concentration)	Final concentration	Volume
Tris-HCl pH 8.0 (50 mM, Recipe 5)	50 mM	1,099 µL
EDTA (10 mM)	1 mM	140 µL
Triton X-100	0.5% (v/v)	7 µL
Guanidine HCl (8 M)	0.8 M	140 µL
***Add before use:***		
Proteinase K	1:100 (8 units/mL)	14 µL
Total	n/a	1.4 mL

**Laboratory supplies**

1. High-performance cover glasses 22 × 22 mm (D = 0.17 mm) (Carl Zeiss, catalog number: 474030-9020-000)
2. High-performance cover glasses 18 × 18 mm (D = 0.17 mm) (Carl Zeiss, catalog number: 474030-9000-000)
3. Scalpels
4. Spoons or spoon spatulas (stainless steel)
5. Scissors
6. Tweezers
7. Pasteur pipettes
8. Caliper
9. Hex nut, 9 mm in diameter
10. Circular glass coverslip, 1.5 mm thickness, 13 mm in diameter (Marienfeld, VWR, catalog number: MARI0117530)
11. Cell culture Petri dishes, non-treated, 35 mm in diameter
12. Cell culture Petri dishes, non-treated, 60 mm in diameter
13. Cell culture 6-well plates
14. Parafilm cut to 18 mm × 18 mm pieces to coat cover glasses of similar size
15. Cyanoacrylate super glue for gluing parafilm to cover glasses
16. In-house 3D-printed spacers with 350 µm thickness [acrylonitrile butadiene styrene (ABS) plastic], see specification of design for 3D printing from Mäntylä et al. [1]
17. In-house 3D-printed molds for cutting the gels [polylactic acid (PLA) plastic], see specification of design for 3D printing from Mäntylä et al. [1]
18. Coverslip cell chamber (Aireka Cells, catalog number: SC15022)
19. 1.5 mL Eppendorf tubes
20. 2.0 mL Eppendorf tubes
21. P1000 pipette tips
22. P200 pipette tips
23. P10 pipette tips
24. Polystyrene box (size L30 cm × H30 cm × W30 cm)
25. Tinfoil
26. Ice

## Equipment

1. Vacuum degassing chamber (e.g., Applied Vacuum Engineering, model: DP3, 3 L)
2. Vacuum pump
3. Microwave
4. Inverted laser scanning confocal fluorescence microscope (e.g., Nikon Instruments, model: Nikon A1R in Ti2 Eclipse)

## Software and datasets

1. Fiji/ImageJ image processing package (open source), Fiji (ImageJ, https://imagej.net/software/fiji/) can be used to analyze acquired microscopy images [9])
2. Noise estimation, denoising, and deblurring (NEDD) software (open source), MATLAB (v1.0.1, release date Apr 17, 2023). The code has been deposited to GitHub: https://github.com/lucioazzari/NoiseEstimationDenoisingDeblurring

## Procedure

- **Iterative indirect immunostaining of samples**
  1. Grow cells to the desired confluency on a 22 × 22 glass coverslip placed in a 6-well plate with 2 mL of growth medium per well.
*Note: Prior to culturing the cells, sterilize coverslips by rinsing three times with dH_2_O and three times in absolute EtOH and air dry for 20 min under UV light inside a laminar hood. Follow cell line–specific instructions in seeding and culturing the cells. If required, coverslips can be protein-coated to enhance cell attachment to the coverslip.*
  2. To fix the sample, add 20% PFA to a final concentration of 4% directly into the medium (400 µL in 2 mL of medium) in a laboratory fume hood. Tilt for an even spread and incubate for 10 min at RT. After fixation, discard the PFA-containing medium and wash the sample two times with 2 mL of 1× PBS (with Ca^2+^/Mg^2+^). Leave in 2 mL of 1× PBS.
  3. Proceed to iterative indirect immunostaining (IT-IF) by performing the first iteration:
*Note: The first iteration corresponds to traditional immunostaining.*
    - Permeabilize the sample for 10 min at RT with 1 mL of the permeabilization buffer A (see Recipes).
    - Dilute the primary antibody/antibodies (Ab/Abs) of choice according to the manufacturer’s instructions in the antibody dilution solution containing 3% BSA as the blocking reagent. Use the recommended and optimized concentrations.
*Note: Careful optimization of the antibody concentration/dilution is required before proceeding to iterative immunostaining.*
    - Transfer the sample-containing coverslip to a clear 6-well plate/35 mm dish for immunostaining by using tweezers. Carefully dip dry the sample on paper to remove excess buffer before adding the primary Ab to avoid uneven spread and dilution of the primary Ab.
    - Add 100 µL of the primary Ab solution to the coverslip and incubate for 1 h at RT.
    - After primary Ab incubation, wash once with permeabilization buffer A, once with PBS, and again with the permeabilization buffer A (each wash with 1 mL for 10 min at RT). Use tweezers to dip dry samples into paper and move them to a clean 6-well plate/35 mm dish for the next staining step.
    - Prepare for the secondary staining by diluting the species-specific and cross-reacted Alexa Fluor 488 -conjugated goat anti-mouse secondary Ab 1:200 to the Antibody diluent solution containing 3% BSA. Protect from light.
    - For secondary staining, add 100 µL of the secondary Ab solution to the coverslip and incubate for 1 h at RT in the dark.
    - After secondary Ab incubation, wash the sample once with the permeabilization buffer A for 10 min, with 1× PBS for 10 min, and again with permeabilization buffer A for 10 min at RT (1 mL each) in the dark.
    - For iterating the staining, repeat primary and secondary immunostaining as described in steps A3b–i four times (four-time iteration) using the same Ab concentration.
*Note: Use permeabilization buffer B (see Recipes) for all the subsequent permeabilization steps in iterations 2–4. Permeabilization buffer B contains a reduced concentration of Triton X-100 (0.2%).*
    - After the washes following iteration 4, wash the sample once in PBS and once in dH_2_O (10 min at RT in the dark). Proceed to anchoring the preceding polymer gel casting.
  For an alternative post-gelling immunostaining technique, see General note 1.
- **Anchoring treatment**
  1. Prepare the anchoring solution (see Recipes).
  2. Place the sample on a fresh 6-well plate.
  3. Pipette approximately 150–200 µL of the anchoring solution on the coverslip containing the cell sample.
  4. Incubate the sample for > 6 h at RT.
*Note: This reaction can be left overnight.*
  5. Wash 2 × 15 min with 2 mL of 1× PBS before proceeding to gelation. Samples can be stored at 4 °C in PBS in the dark before proceeding with the protocol.
- **Gelling**
*Note: Tools used in this section are presented in Figure 1.*
  1. Cut parafilm to a slightly larger size than the glass coverslip (18 mm × 18 mm) and do not remove the covering paper. Add one drop of glue to the coverslip and stick the parafilm to the glue with its paper-covered side facing up. Press gently and let dry. Since the gel does not adhere to parafilm, the parafilm-mounted coverslip will be used as the bottom of the gelation chamber. See Figure 1A showing the parafilm-mounted coverslip upside down to demonstrate the respective sizes of the parafilm and the coverslips after gluing.
*Note: The parafilm prevents the gels from sticking to the top glass slide.*
  2. With the covering paper facing up, place the parafilm-mounted coverslip on a 6-well plate lid/60 mm Petri dish and remove the covering paper to expose the clean parafilm surface on the coverslip (Figure 1B).
  3. Place the in-house 3D-printed spacer (Figure 1C) on the exposed parafilm surface of the coverslip (Figure 1D).
*Note: This spacer controls the circular shape, size (opening r = 5 mm), and height (350 µm) of the polymer gel in the gelation phase. The size of the polymerized gel is required for calculating the expansion factor after the expansion. By using the in-house printed 3D spacers, the size of the gel is always constant and equals the size of the spacer opening. However, the size of the gel can also be measured using a caliper.*
  4. Cool a new 2 mL Eppendorf tube on ice.
  5. Prepare Gelling solution (see Recipes) in the 2 mL Eppendorf placed on ice.
*Note: Do not add initiator (APS) yet! One can prepare two samples from one gelling solution aliquot at the same time.*
  6. Once ready with the samples, add 10% APS (Recipe 7) to the gelation solution, vortex briefly, and immediately pipette 70 µL of gelation solution into the middle of the opening of the 3D-printed spacer placed on the parafilm-mounted coverslip (Figure 1E). The spacer controls the circular shape, size (opening r = 5 mm), and height (350 µm) of the polymer gel in the gelation phase.
*Note: Pipetted volume depends on the sample size, so you might need to scale this if, e.g., tissue samples are being used.*
  7. Carefully but rapidly place the sample coverslip containing the immunolabeled cells on top of the gelling solution droplet with cells facing down, i.e., toward the droplet of gelling solution. The cells will be inside the gel after polymerization.
*Note: Remove any extra PBS from the sample coverslip by touching it with tissue paper. This prevents PBS from diluting the gel solution.*
  8. Add a metal hex nut on top of the sample (Figure 1F).
  9. Protect the sample from light by covering them, e.g., with tinfoil, and let the gel polymerize for 30–45 min at RT.
  10. After polymerization, carefully remove the hex nut and flip the sample (sandwiched coverslips containing the spacer and the polymerized gel in between) so that after flipping, the parafilm-mounted coverslip is on top and the sample-containing coverslip is at the bottom. Remove the parafilm-mounted coverslip by gripping the parafilm edge with tweezers. Discard the parafilm-mounted coverslip. Remove the spacer. A circular gel remains on the sample coverslip with cells located at the bottom (facing the sample coverslip). If needed, use a scalpel to cut and remove any excess gel.
*Note: The expansion will increase the gel size by approximately 4×, thus gels over 1 cm will be difficult to handle.*
  11. Place the gel (still attached to the sample coverslip) into a fresh well of a 6-well plate with the gel facing up.
  12. The diameter of the polymerized gel is required for calculating the expansion factor after the expansion. By using the in-house-printed 3D spacers with known dimensions, the diameter of the gel is always constant and equals the diameter of the spacer opening (d = 10 mm). However, the diameter or some other size-related parameter of the gel can also be measured after the polymerization by using a caliper, especially if a spacer has not been used for casting the gel (Figure 1L). The expansion factor (equation described in section E) is required for pixel size quantification of the microscopy images.
*Note: The gel will start to swell during the next digestion step; thus, it is crucial to measure the original gel size before that step.*
    **Figure 1.**
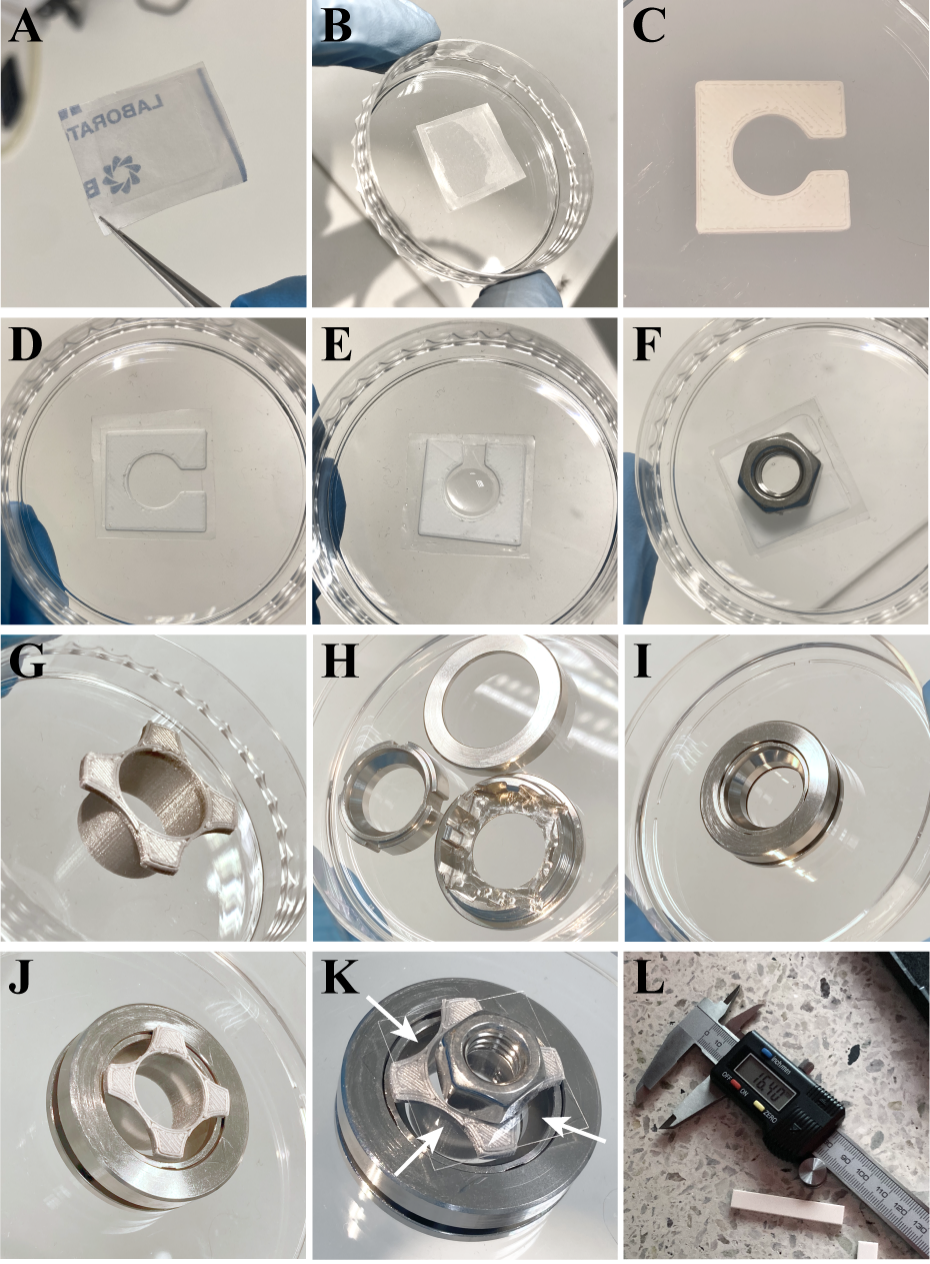
Tools used in gel casting, mounting for imaging, and measuring the expanded gel. A. Parafilm piece glued to an 18 mm × 18 mm glass coverslip with super glue (turned upside down to demonstrate respective dimensions of the cut parafilm and the coverslip). B. Parafilm-coated coverslip in a Petri dish with the cover removed. C. In-house 3D-printed plastic gel casting spacer. D. Gel casting spacer placed on a parafilm-coated 18 mm × 18 mm glass coverslip. E. Drop of gelling solution in a gel casting spacer placed on a parafilm-coated 18 × 18 glass coverslip. F. Metal hex nut placed on top of the cell-containing 22 × 22 coverslip placed upside down into the gelling solution pipetted to a gel casting spacer on an 18 × 18 coverslip shown in E. G. In-house 3D-printed plastic gel cutting mold. H. Coverslip cell chamber, unassembled. I. Assembled coverslip cell chamber with a 22 × 22 glass coverslip on the bottom. J. Gel cutting mold containing a gel placed into the coverslip cell chamber containing a 22 × 22 coverslip. K. Sample preparation is finished by adding water on top of the gel to prevent drying and placing another 22 × 22 coverslip with a metal hex nut on top as a weight to keep the assembly in place. Arrows represent places where molten high-melt agar should be pipetted to mount the assembly prior to imaging. L. An example of a digital caliper used to measure the PAA-hydrogel after isotropic expansion in water to determine the expansion factor before proceeding to the gel cutting and mounting to the coverslip cell chamber.
- **Digestion**
  1. Prepare the digestion solution (see Recipes).
  2. Digest the polymerized gel containing the cell sample on a 60 mm Petri dish with the digesting solution by adding at least 10-fold excess volume of digestion buffer (e.g., ~2 mL) overnight (12 h) in the dark at RT.
  3. During the digestion, the gel with the cells inside will detach from the sample coverslip. The coverslip can remain in the Petri dish, but it can also be discarded.
*Note: Do not peel the gels off the surface of the glass as this will damage the sample!*
  4. After digestion, move the polymerized gel by using a spoon/spatula to a fresh 60 mm Petri dish.
*Note: If the coverslip is already detached, you can remove it from under the sample and discard it.*
- **Expansion**
  1. Expand the gel sample isotropically by adding an excess volume of dH_2_O (e.g., 20 mL/60 mm dish). Incubate for 1 h in the dark at RT.
*Note: Use at least 10× the final gel volume.*
  2. Discard water carefully and replace it with fresh water.
*Notes:*
    - *The gel has the same refractive index as water. Use caution when removing the water. Avoid vacuum aspiration.*
    - *Gel expansion reaches a plateau after the third or fourth wash.*
  3. Repeat steps E1–2 at least five times.
  4. After removing the water for the last time, measure the diameter (or other size-related parameters as described in step C12) of the expanded gel with a caliper (Figure 1L).
  5. Calculate the expansion factor using the following equation.
$$Expansion\;factor=\frac{Gel\;diameter\;post\;expansion}{Gel\;diameter\;prior\;expansion}$$
*Note: By casting the gel in the opening (r = 5 mm, height 350 µm) of the in-house printed 3D spacer, a circular gel with known dimensions will be produced. The diameter of the spacer opening can be used as the pre-expansion gel diameter. After the expansion of the polymerized gel, measure the expanded gel diameter and use that as a post-expansion gel diameter to calculate the expansion factor. For traditional ExM, the expansion factor is near 4. Use this factor to determine the real size of your objects, for example during image analysis as described below in Data analysis section.*
- **Sample mounting and microscopy**
*Note: The tools used in this section are presented in Figure 1.*
  1. After the isotropic expansion in water, cut a circular piece of the expanded gel with a 3D-printed mold (Figure 1G). Put the gel with the mold still attached on a new 22 mm × 22 mm coverslip. Place it inside the Aireka coverslip cell chamber (Figure 1H–J).
  2. Place a 13 mm diameter glass coverslip on top of the gel and add a 9 mm hex nut to the top to keep the gel in place during mounting in the next step (Figure 1G–K).
  3. Mount the gel-containing mold in the coverslip cell chamber with melted 1% high-melt agarose. **Caution:** Hot agarose is a burn hazard!
*Note: A volume of 50 mL of high-melt agarose can be melted in a microwave in a glass bottle with a cap only loosely placed on top. Use 800 W power in 30 s intervals and swirl the bottle gently between the intervals. Avoid bubbles. Repeat until completely liquid and clear. Avoid overheating or boiling the agarose in the microwave. Let cool down at RT for 5–10 min. Add slightly cooled melted agarose (≤ 1 mL) to the empty sides between the mold and the chamber (not inside the mold/on top of the sample!) by pipetting with a Pasteur pipette. The agarose should not enter under the gel.*
  4. Add dH_2_O on top of the sample inside the mold to prevent the sample from drying and shrinking. The coverslip and the nut can be left in place and kept there during imaging to ensure that the sample stays in place to avoid drift.
  5. The Aireka coverslip cell chamber fits in a regular 35 mm dish sample holder on a confocal microscopy stage. After placing it, let the temperature stabilize before the imaging. After 15–30 min, acquire images according to your needs.
*Note: Imaging can be done according to the analysis requirements.*
  6. For imaging, use a water immersion (WI) objective with a high working distance and numerical aperture (N.A.) such as Nikon CFI Plan Apo IR SR 60× WI, N.A. 1.27, with a working distance of 0.17 mm. To acquire images for quantitative analysis, record an optical z-section series of 1,024 × 1,024 pixels (or similar) with a pixel size ≤ 50–70 nm in the x- and y-directions and ≤ 200 nm in the z-direction for optimal resolution and pixel size for mathematical deconvolution or signal processing.
- **NEDD denoising**
  Before analyzing the data, our open-access signal processing software NEDD (MATLAB) is recommended to further improve the signal-to-noise ratio of the images acquired from samples prepared with the above protocol for ExM. Our NEDD pipeline in MATLAB assumes the noise to be additive, spatially correlated, and with signal-dependent variance [10].
*Note: The NEDD can process any LSCM image data and is not restricted to ExM.*
  The pseudo-algorithm of the NEDD pipeline can be summarized as follows:
  1. Estimate the noise variance and power spectral density (PSD) of the data.
  2. Apply a variance stabilizing transform (VST) to the data using the noise parameters estimated in the previous step. We use the generalized Anscombe transform [11].
  3. Denoise the stabilized data using the iterative framework introduced in [12]. The denoising filter used within the framework is the RF3D algorithm designed for processing videos (three-dimensional data) corrupted by correlated noise [13].
  4. Apply the inverse VST to return the data to its original intensity domain.
  5. OPTIONAL: Perform deconvolution (deblurring) of the denoised sequence using a regularized Tikhonov deconvolution approach [14].
  The software comes with a demo (*demo.m*), that can be run to demonstrate its usage, which can be called with the command:
  noisy,denoised,deblurred]=processData(inputPath,outputPath,noiseModel,optionalParams)
  where the inputs are:
  - *inputPath*: A string containing the path to the input noisy file. The file must be in single-channel gray-scale multi-page TIFF format.
  - *outputPath:* A string containing the path where to store the single-channel gray-scale multi-page TIFF output.
  - *noiseModel*: A string that defines the noise model to be assumed for estimation and denoising. It can be either *white* or *colored.*
  - *optionalParams:* A MATLAB struct variable that contains optional parameters that can be used to adjust, e.g., the denoising filter and the deblurring parameters. More details can be found in the function’s help.

The function will write the processed data into the *outputPath.* Additionally, the function returns to the workspace:

- *noisy*: The noisy data (M × N × F double array) read from *inputPath.*
- *denoised*: The denoised data (M × N × F double array).
- *deblurred*: The deblurred data (M × N × F double array)

## Data analysis

The application of LSCM in the imaging of ExM gels enables detailed quantitative image analyses, although the resolution is limited by diffraction. The traditional expansion leads to 4–6× enlargement of the samples, enabling visualization beyond the traditional diffraction limit of light microscopy. When performing image analysis or visualization, remember to consider the expansion factor in the pixel size or in the scale bar.

*Note: To acquire the correct image scale, divide the voxel dimensions with the expansion factor.*

The watery expansion of the gel will induce isotropic expansion, where the sample enlarges in all three dimensions. Thus, a cell nucleus of 20 µm in x/y diameter and 5 µm in z will appear a lot larger, approximately 40–120 µm in x/y and 20–30 µm in vertical direction. This results in long imaging times, where the sample needs to stay in place. However, if any movement occurs during imaging, it can be mathematically corrected in analysis programs. Moreover, the long imaging times might result in bleaching of the sample, as high laser powers are traditionally needed in ExM imaging. This is because the expansion dilutes the antibody-conjugated fluorophores in the sample, reducing the resulting image brightness, where the dilution scales to the third power of the (linear) expansion factor, and thus even 4× expansion can lead to a 64× reduction of the fluorescent signal. The IT-IF technique overcomes this traditional challenge in ExM and induces higher labeling density, leading to significantly improved signal intensity without compromising the SBR, advancing super-resolution imaging of highly compacted and proteinaceous intranuclear structures.

For the quantitative analysis, ensure you have at least three biological replicate samples, and image ~10–20 cells each, depending on the study. Also, due to the sensitivity of the technique and to reduce the statistical variance between the samples, always use predetermined and identical volumes when adding antibodies, washing, or otherwise treating the samples. Any errors or exceptions in the treatment protocol are likely to affect the intensity and SBR of the sample and cause differences between technical replicates of the sample.

To perform analyses, any open-source and free image analysis software such as Fiji/ImageJ [8] can be used. In analyzing the data, you can, for example, measure the total intensity of the sample by first making a maximum intensity z-projection, intensity thresholding and measurement of the region of interest, and normalizing it by subtracting the background intensity measured outside your target.

**Figure 2.**
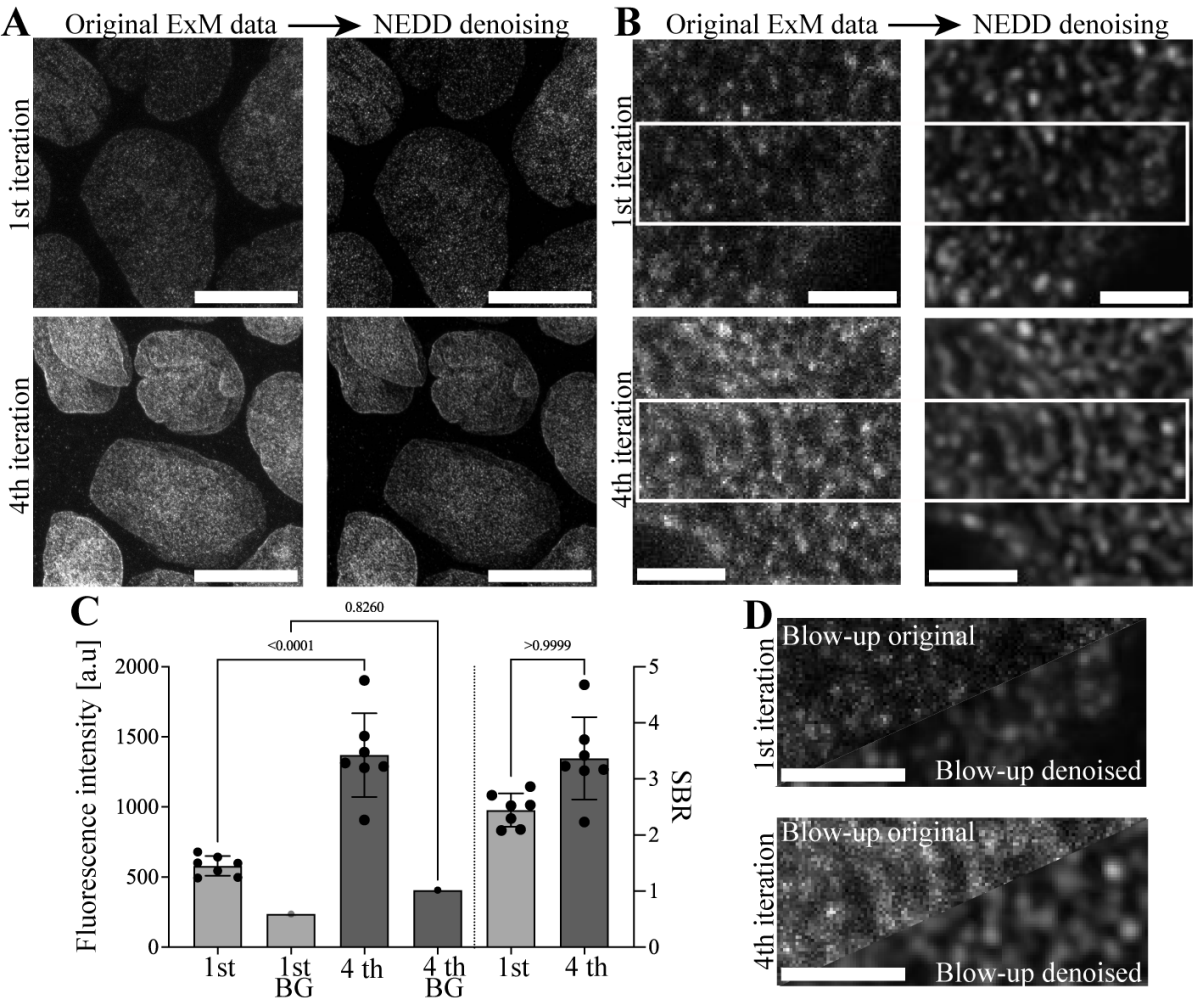
Results of nuclear lamin A/C detection in noise estimation, denoising, and deblurring (NEDD)-denoised expansion microscopy–laser scanning confocal microscopy (ExM-LSCM) images following either traditional (1×) or iterative indirect immunostaining (IT-IF). A. Representative grayscale maximum projection LSCM images of nuclear lamin A/C of epithelial Madine Darby canine kidney type II (MDCKII) cells after the first iteration of corresponding traditional (1×) immunostaining (upper panels) and 4× iterated (fourth iteration) indirect immunostaining (IT-IF, lower panels) before (left panels) and after signal denoising (NEDD, right panels). Scale bars, 10 µm. B. Blow-out images showing details of lamin A/C following first and fourth iterations and NEDD denoising. Scale bars, 1 µm. C. Quantification of the fluorescence intensity (left y-axis) and signal-to-background ratio (SBR) (right y-axis) of nuclear lamin A/C and the background after first and fourth iterations. Error bars represent the standard deviation of the mean. One-way ANOVA was used to test for statistical significance. D. Collective blow-up images of the NEDD-denoised lamin A/C IT-IF data after first (upper panel) and fourth iteration (lower panel) from a region of interest indicated by a white box in B for direct comparison. Scale bars, 1 µm.

In Figure 2, we showcase the power of the presented pro-ExM protocol and the analysis of our published NEDD-denoised IT-IF-ExM-LSCM data generated using this protocol for visualization of lamin A/C organization in epithelial cells by using traditional (1×) immunostaining and IT-IF with four iterations (from first to fourth iteration) [1].

The results show how IT-IF combined with ExM-LSCM and NEDD denoising substantially improve the visualization of lamin A/C organization (Figure 2A, 2B, and 2D). We found that the IT-IF pro-ExM protocol results in significantly higher intensity and SBR of the lamin A/C (Figure 2C), and NEDD-denoising further improves the detection of lamin organization in high resolution (Figure 2A, 2B and 2D). Thus, our IT-IF and NEDD protocol provides a significant improvement to the detection and quantification of the structural organization of nuclear lamina in ExM.

## Validation of protocol

This protocol or parts of it has been used and validated in the following research articles:

Iterative immunostaining and denoising protocol:

- Mäntylä et al. [1]. Iterative immunostaining combined with expansion microscopy and image processing reveals nanoscopic network organization of nuclear lamina. *Molecular Biology of the Cell* (Figure 2, panels G–I; Figures 3–5).

Protein retention expansion microscopy protocol:

- Tillberg et al. [15]. Protein-retention expansion microscopy of cells and tissues labeled using standard fluorescent proteins and antibodies. *Nature Biotechnology* (Figures 1–3).

## General notes and troubleshooting

**General notes**

1. The iterations included in the protocol can be done subsequently without pauses or by dividing the work for four subsequent days (one iteration per day). For the latter, store the samples in 1× PBS at 4 °C overnight in between the iterations. This might be beneficial to enhance washing.
2. Immunostaining can also be done post-gelling. However, consider that this will require higher reagent volumes and concentrations of antibody solutions and longer incubation times as the diffusion of subjects across the gel takes time.
3. In a traditional proExM protocol, 4× concentrations of the primary Ab and 2× concentrations of the secondary Abs in comparison to normal immunofluorescence staining are used. Here, carefully optimized 1× concentrations apply.

**Troubleshooting**

Problem 1: Gel does not polymerize.

Possible causes: APS or TEMED is not working, or sodium acrylate solution might be expired or not pure.

Solutions: Prepare fresh 10% (w/v) APS. 10% (w/v) APS should be aliquoted and stored in a desiccator at -20 °C, as APS is hygroscopic. TEMED is also highly hygroscopic and accumulated water leads to its decomposition. You can also use parafilm to cover the aliquoted tube caps to prevent moisture from getting into the tube.

Problem 2: Gel is not attaching to the cells and/or part of the sample remains attached to the coverslip.

Possible cause: Crosslinking by using the anchoring solution is incomplete.

Solutions: Extend the anchoring time or prepare a fresh anchoring solution. Acryloyl-X is a succinimidyl ester-based linker. In anchoring, PFA-fixed and immunostained molecules undergo amine-acryloyl conversion to enable their covalent anchoring to the polymeric gel. Make sure that you use only clear Acryloyl-X solutions. Pure sodium acrylate solution is clear, whereas contaminated solution will have a yellow tint. Store Acryloyl-X as undiluted aliquots. Succinimidyl ester decomposes rapidly in water, especially in pH > 7; thus, use dry and fresh DMSO to prepare the stock solutions (DMSO is hygroscopic).

Problem 3: Gel is polymerizing too quickly, e.g., during pipetting.

Possible causes: Too high accelerator (TEMED) and/or initiator (APS) concentrations.

Solutions: Work in a laboratory with a less moist atmosphere. Prepare gelling solutions with less TEMED/APS. Make sure you keep all the solutions and tubes on ice. Add APS only before pipetting to the sample. If the gel is still polymerizing too quickly, replace water with 4-Hydroxy-TEMPO. This will slow down the reaction but not prevent it.

Problem 4: Only parts of the gel expand during expansion in water.

Possible cause: Uneven gelling solution following inadequate mixing.

Solution: Mix your gelling solution thoroughly before adding APS.

Problem 5: Gel drifts during LSCM imaging.

Possible causes: The sample is not mounted properly, or the temperature is higher during imaging causing movement of the agar used for mounting.

Solution: Apply only high-melt agar for mounting the samples. Keep samples under a weight (glass coverslip and a hex nut) during imaging. Check the temperature of the microscopy room/incubator. Imaging should be done at RT and in stable conditions. Let the sample stabilize on the microscopy stage for at least 15 min before imaging.

Problem 6: Antibody background is too high after iterations.

Possible causes: Poor quality of secondary antibodies or off-target binding of the primary antibody.

Solution: Carefully optimize the antibody concentration before iterations. Iteration works for most of the antibodies. However, polyclonality might present challenges. Try another and prefer monoclonal antibodies.

## References

[1] Mäntylä E., Montonen T., Azzari L., Mattola S., Hannula M., Vihinen-Ranta M., Hyttinen J., Vippola M., Foi A., Nymark S., .(2023). Iterative immunostaining combined with expansion microscopy and image processing reveals nanoscopic network organization of nuclear lamina. Mol Biol Cell. 34(9): ee22–09–0448. 10.1091/mbc.E22-09-0448 PMC10398900 37342871

[2] Truckenbrodt S., Maidorn M., Crzan D., Wildhagen H., Kabatas S. and Rizzoli S. O. (2018). X10 expansion microscopy enables 25‐nm resolution on conventional microscopes. EMBO Rep. 19(9): e201845836. 10.15252/embr.201845836 PMC6123658 29987134

[3] Zhao Y., Bucur O., Irshad H., Chen F., Weins A., Stancu A. L., Oh E. Y., DiStasio M., Torous V., Glass B., .(2017). Nanoscale imaging of clinical specimens using pathology-optimized expansion microscopy. Nat Biotechnol. 35(8): 757-764. 28714966 10.1038/nbt.3892 PMC5548617

[4] Lau L., Lee Y. L., Sahl S. J., Stearns T. and Moerner W. (2012). STED Microscopy with Optimized Labeling Density Reveals 9-Fold Arrangement of a Centriole Protein. Biophys J. 102(12): 2926-2935. 22735543 10.1016/j.bpj.2012.05.015 PMC3379620

[5] Whelan D. R. and Bell T. D. M. (2015). Image artifacts in Single Molecule Localization Microscopy: why optimization of sample preparation protocols matters. Sci Rep. 5(1): e1038/srep07924. 10.1038/srep07924 PMC4300460 25603780

[6] Ihalainen T. O., Aires L., Herzog F. A., Schwartlander R., Moeller J. and Vogel V. (2015). Differential basal-to-apical accessibility of lamin A/C epitopes in the nuclear lamina regulated by changes in cytoskeletal tension. Nat Mater. 14(12): 1252-1261. 26301768 10.1038/nmat4389 PMC4655446

[7] Schnell U., Dijk F., Sjollema K. A. and Giepmans B. N. G. (2012). Immunolabeling artifacts and the need for live-cell imaging. Nat Methods. 9(2): 152-158. 22290187 10.1038/nmeth.1855

[8] Chen J., Sasaki H., Lai H., Su Y., Liu J., Wu Y., Zhovmer A., Combs C. A., Rey-Suarez I., Chang H. Y., .(2021). Three-dimensional residual channel attention networks denoise and sharpen fluorescence microscopy image volumes. Nat Methods. 18(6): 678-687. 34059829 10.1038/s41592-021-01155-x

[9] Schindelin J., Arganda-Carreras I., Frise E., Kaynig V., Longair M., Pietzsch T., Preibisch S., Rueden C., Saalfeld S., Schmid B., .(2012). Fiji: an open-source platform for biological-image analysis. Nat Methods. 9(7): 676-682. 22743772 10.1038/nmeth.2019 PMC3855844

[10] Azzari L., Borges L. R. and Foi A. (2018). Modeling and estimation of signal-dependent and correlated noise. In: Bertalmío, M.(Ed.). *Denoising of Photographic Images and Video*. Fundamentals, Open Challenges and New Trends.(pp. 1–36). Advances in Computer Vision and Pattern Recognition. Springer.

[11] 11. Starck J. L., Murtagh F. D. and Bijaoui A. (1998). Image processing and data analysis: the Multiscale approach. Cambridge University Press.

[12] Azzari L. and Foi A. (2014). Indirect Estimation of Signal-Dependent Noise With Nonadaptive Heterogeneous Samples. IEEE Trans Image Process. 23(8): 3459-3467. 24808411 10.1109/TIP.2014.2321504

[13] Maggioni M., Sanchez-Monge E. and Foi A. (2014). Joint Removal of Random and Fixed-Pattern Noise Through Spatiotemporal Video Filtering. IEEE Trans Image Process. 23(10): 4282-4296. 25122566 10.1109/TIP.2014.2345261

[14] Dabov K., Foi A., Katkovnik V. and Egiazarian K. (2008). Image restoration by sparse 3D transform-domain collaborative filtering. SPIE Proceedings: e766355. 10.1109/tip.2007.901238 17688213

[15] Tillberg P. W., Chen F., Piatkevich K. D., Zhao Y., Yu C. C., English B. P., Gao L., Martorell A., Suk H. J., Yoshida F., .(2016). Protein-retention expansion microscopy of cells and tissues labeled using standard fluorescent proteins and antibodies. Nat Biotechnol. 34(9): 987-992. 27376584 10.1038/nbt.3625 PMC5068827

